# Wearable Sensor Based Stooped Posture Estimation in Simulated Parkinson’s Disease Gaits

**DOI:** 10.3390/s19020223

**Published:** 2019-01-09

**Authors:** Quoc Khanh Dang, Han Gil Seo, Duy Duong Pham, Youngjoon Chee

**Affiliations:** 1 Impedance Imaging Research Center (IIRC), Kyung Hee University, Seoul 02447, Korea; khanhdq8689@gmail.com; 2 Department of Rehabilitation Medicine, Seoul National University College of Medicine, Seoul National University Hospital, Seoul 03080, Korea; hgseo80@gmail.com; 3 Electrical and Electronic Engineering department, The University of Danang—University of Technology and Education, Danang 550000, Vietnam; duyduongd2@gmail.com; 4 School of Electrical Engineering, University of Ulsan, Ulsan 44610, Korea

**Keywords:** stooped posture, Parkinson’s disease, accelerometer

## Abstract

Stooping is a posture which is described as an involuntary forward bending of the thoracolumbar spine. Conventionally, the stooped posture (SP) in Parkinson’s disease patients is measured in static or limited movement conditions using a radiological or optoelectronic system. In the dynamic condition with long movement distance, there was no effective method in preference to the empirical assessment from doctors. In this research, we proposed a practical method for estimating the SP with a high accuracy where accelerometers can be mounted on the neck or upper back as a wearable sensor. The experiments with simulated subjects showed a high correlation of 0.96 and 0.99 between the estimated SP angle and the reference angles for neck and back sensor position, respectively. The maximum absolute error (0.9 and 1.5 degrees) indicated that the system can be used, not only in clinical assessment as a measurement, but also in daily life as a corrector.

## 1. Introduction

Stooped posture (SP) is one of the main appearances of Parkinson’s disease (PD) [1,2]. SP is described as an involuntary forward bending of the thoracolumbar spine, and is mostly found in movement disorders. A serious survey and analysis conducted by Margraf et al. [3] reported that SP had caused back pain in 86% of PD patients. This research also noted that SP led to burdens in life, since 77% of PD patients needed to use walking aids and 85% experienced specific disabilities, such as dyspnea and fall risk. As neck flexion and fore-bent angles were seen to have a high correlation and were associated with PD duration [4], SP also reduces the ability of PD patients to look forward. Furthermore, late diagnosis may lead SP to become rigid kyphosis, which is much more challenging to correct [5].

To date, the pathophysiology of SP in PD is not well understood. For decades, a considerable amount of literature has been published on the Parkinsonism SP treatment. However, no consensus method has been accepted [6]. Proposed remedies can generally be classified into surgical, pharmacological, and non-pharmacological modalities. Despite the fact that surgical intervention showed some benefits in posture correction [7], the procedure was complicated to apply to all patients. Thus, surgical approaches are only considered as an alternative to pharmacological methods. Pharmacological approaches take the effect of using drugs such as levodopa [8], botulinum neurotoxin [9], and lidocaine [10] to relieve SP symptoms. However, there has been no clear correlation between these medical treatments and SP, since their efficacies were reported to be controversial or unpredictable. Different from surgical and pharmacological methods, the non-pharmacological approach has been considered preferable, thanks to its simplicity and potency. A number of studies examining the influence of using postural orthotic wearables on SP treatment showed that SP improved and back pain lessened significantly [11,12]. A large-scale report in Reference [5] found that the average pain score reduced by 70% and the quality of life increased 92% after using a thoraco-pelvic anterior distraction orthosis for 90 days. Subsequently, Tomlinson et al. [13] concluded that physiotherapy could develop the gait and balance of PD patients. In addition, the PD patients in Reference [1] were able to voluntarily and temporarily straighten up when asked to stand upright. These conclusions drove us to the idea of alleviating SP using a wearable device, which can measure and help patients correct their posture by providing a reminder when they are in a SP, even while walking.

To the best of our knowledge, there have been no studies on using wearable sensors to measure the severity of SP in PD while walking. The use of the inertial sensors in evaluating angular kinematic of lower limbs during gait for both healthy people [14,15,16] and PD patients [17,18] has a long history, but there has been no consideration for SP. Conventionally, SP in PD can be measured using radiological or optoelectronic systems [19]. Radiological measurements are a gold standard for evaluating spinal deformities. Despite its precision, the radiological method suffers from a major drawback due to its harmful radiological exposure. Alternatively, optoelectronic measurement can be used. The optoelectronic system provides a non-invasive and accurate measurement in both static and dynamic conditions [20,21]. Yet the working area is still limited in the range of view of the capturing devices. That limitation brought a hindrance to quantitatively assessing the severity of SP in PD, especially under dynamic conditions because SP is gradually aggravated during walking in most patients with PD. For example, in a six-minute walking test, where patients were asked to walk back and forth on a long straight path, the SP severity of the patients can only be evaluated based on questionnaires and empirical assessments from doctors [22]. Thus, in this study, we propose a novel method utilizing the mobility advantage of wearable sensors to estimate SP in PD, which can help in assessing the severity, even when walking. This research mainly focuses on evaluating the measurement accuracy with the data from simulated subjects to corroborate the feasibility of using wearable sensors in Parkinsonism SP treatment. The wearable sensor consists of a 3-axis accelerometer that acts as an inclinometer. A similar idea was implemented in some posture correction products; for example, ALEX (Namu, Ulsan, Korea) and Upright Go (Upright Technologies, Yehud, Israel). With this concept, in a static pose, the flexion of the trunk or neck can be measured with considerable accuracy. However, measuring SP under dynamic conditions, such as walking, seems to be challenging for these products, due to the noises caused by patient tremors and movements. In this paper, we applied an averaging algorithm and evaluated its efficacy in removing tremor and movement artifacts.

The paper is organized as follows. Section 2 describes the methodology, and the measurement principle and experiment setup are explained in detail. The experiment results are reported in Section 3 and Section 4 summarizes the work with some discussions and implications for future work. Finally, a brief summary and critique of the findings are given in Section 5.

## 2. Methods

### 2.1. Measurement Principle

In a previous study conducted by Siminoski et al., the trunk’s flexion angle was indirectly measured by the horizontal distance between the C7 and S1 bones (C7-SAR distance) [23]. The principle was motivated by the use of radiological and optoelectronic systems, where the kyphosis angle could be measured directly since the positions of the C7 and S1 bones are explicit. The result in [23] showed that the kyphosis angle and C7-SAR distance were correlated, and the C7-SAR distance can be used as an indirect method in clinical practice to estimate trunk flexion. In this study, the C7-SAR distance obtained by an OptiTrack Flex 3 system (NaturalPoint, Corvallis, OR, USA) was used as ground-truth value to compare with the estimated trunk’s flexion angles. The camera system consisted of six infra-red cameras recording at 100 FPS with a maximum 58-degree field of view. Data were recorded using professional motion tracking software, Motive:Tracker (NaturalPoint, Corvallis, OR, USA), which supports 6 DOF ( Degree of Freedom) tracking in real-time.

**Figure 1:**
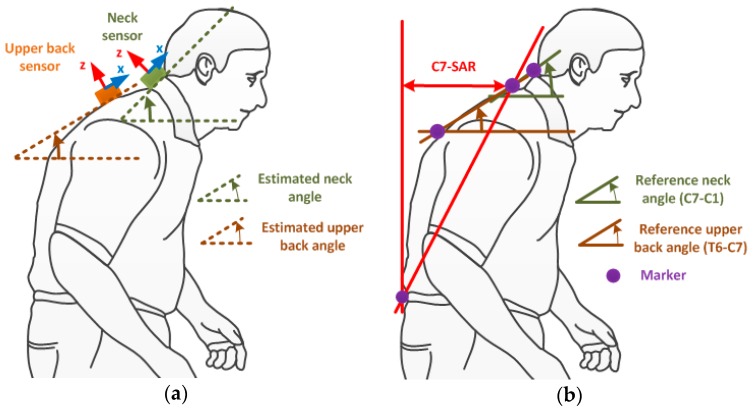
Sensor locations and estimated parameter definitions: (**a**) Sensor locations; (**b**) estimated parameter definitions.

The aim of this study was to clarify the efficacy of implementing a wearable sensor in measuring the severity of SP in PD. The sensor was chosen to be mounted on the upper back (T2 bone) since this position might accurately represent SP. To determine whether the upper back is the preferential position for measuring, we also quantitatively analyzed the neck sensor position at the C4 bone, as neck flexion has a high correlation with the trunk forward bending angle [4]. The estimated SP angles were defined by the inclination angles of the sensors at their positions, while the reference angles for the neck and back flexion were described by the angles formed by the transverse plane and the ($\bar{C_{7}C_{1}}$) and ($\bar{T_{6}C_{7}}$) lines, respectively, as can be seen in Figure 1. 

In the quasi-static pose, the sensor’s inclination angle could be calculated using the accelerometer output at a certain time. Let $a={\left[a_{x},\;a_{y},a_{z}\right]}^{T}$ be the acceleration value obtained from the sensor, the inclination angle α is defined as [24]

(1) $$\alpha =a\tan\frac{a_{z}}{\sqrt{a_{x}^{2}+a_{y}^{2}}}$$

**Figure 2:**
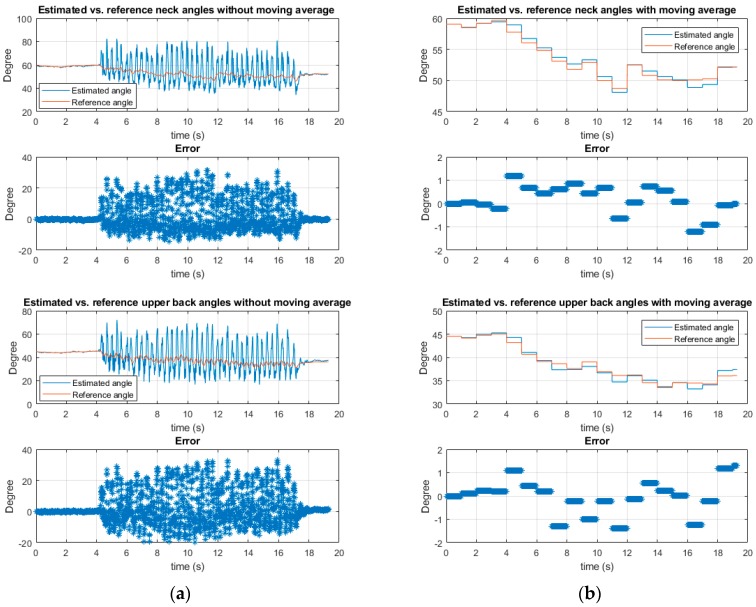
Motion artifacts elimination using moving average method: (**a**) Estimation without moving average. From top to bottom: The comparison between estimated and reference angle of the neck without using moving average method—their relative errors—the comparison between estimated and reference angle of the upper back without using moving average method—their relative errors; (**b**) estimation with a moving average window of 1 s (non-overlapping). From top to bottom: The comparison between estimated and reference angle of the neck using moving average method—their relative errors—the comparison between estimated and reference angle of the upper back using moving average method—their relative errors.

However, the estimation using Equation (1) may be unreliable under dynamic conditions, especially while walking with tremors in PD patients. Although walking and tremor motions are identified as major influencing artifacts, they can be mainly eliminated by using averaging algorithms, owing to their symmetric waveforms. A deep analysis made by Jang et al. [25] reported that tremors in PD are symmetric. In the same vein, human gait has been illustrated to have a sinusoidal form, even at high walking speeds [26]. For the purpose of verification, a pilot test of implementing an averaging algorithm was done with a Parkinsonism simulated subject. A moving average window of one second was applied to the accelerometer data, which were sampled at 100 Hz. The results of a simulated PD gait with a vigorous tremor and festinating gait showed that the estimated errors due to motion artifacts were significantly removed, as can be seen from Figure 2b. The RMSE (Root Mean Square Error) of the estimated inclination angles reduced significantly from 9.01 and 10.25 degrees to 0.62 and 0.72 degrees for the neck and upper back sensor positions, respectively. The maximum absolute errors were also reduced from 31.27 and 33.1 degrees to 1.22 and 1.39 degrees after implementing the moving average filter.

### 2.2. Experiment Setup

**Figure 3:**
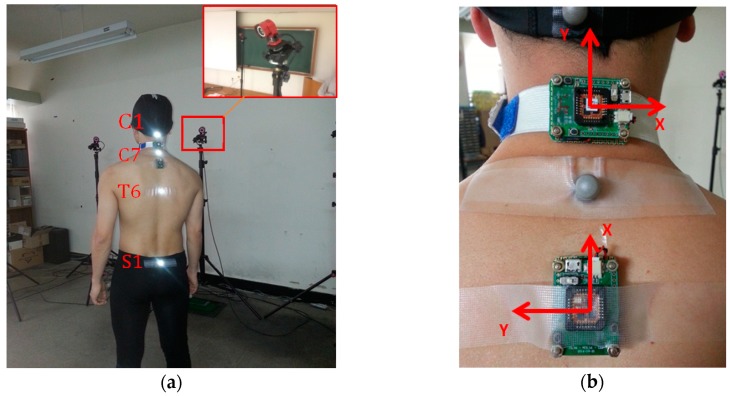
Experiment Setup: (**a**) Camera and marker locations; (**b**) sensor installation and coordinate frames.

Seven male subjects with ages ranging from 24 to 30 were trained to simulate PD gait under the supervision of experts from the Seoul National University Hospital. The subjects were asked to perform SP with a vigorous tremor and shuffling gait while walking on a four-meter-long straight path. Along the path, an OptiTrack camera system with six Flex3 cameras was installed to record the posture data from the walk, as shown in Figure 3a. Four camera markers were mounted on the C1, C7, T6 and S1 bones, respectively, in order to measure the flexion of the neck and upper back, as well as the C7-SAR distance for reference measurements (see Figure 1b). Two wireless inertial sensor boards containing XSens M1 sensors (Xsens Technologies B.V., Enschede, the Netherlands) were installed on the subject’s neck and upper back, as shown in Figure 3b. The accelerometer data from two sensors were stored in Flash memory, to be used later to calculate the flexion of the neck and upper back. We verified the performance of the sensors by comparing the reference data from the camera system and the estimated data from the sensors.

In the experiments, each subject performed four types of walking, as follows:

(1) Slow PD gait while maintaining SP with tremor
(2) Fast PD gait while maintaining SP with tremor
(3) Slow PD gait while changing SP with tremor
(4) Fast PD gait while changing SP with tremor

In (3) and (4), the trunk flexion was increased gradually from the upright posture at the starting point of the walk to the maximum angle that the subject could perform, then gradually recovered to an upright posture at the end point of the walk. Each type of walking was repeated in five trials. At the beginning of each walk, subjects were asked to stand upright for a few seconds in order to calculate the initial inclination angle of each sensor. This was used to calibrate the initial differences between the sensor and camera systems’ coordinate frames. Since the sensor boards can be controlled wirelessly, they were synchronized with the camera system so that the data from both systems were recorded simultaneously. In total, 140 datasets were obtained, where each set contained the neck and upper back posture data for four meters of walking.

## 3. Results

**Figure 4:**
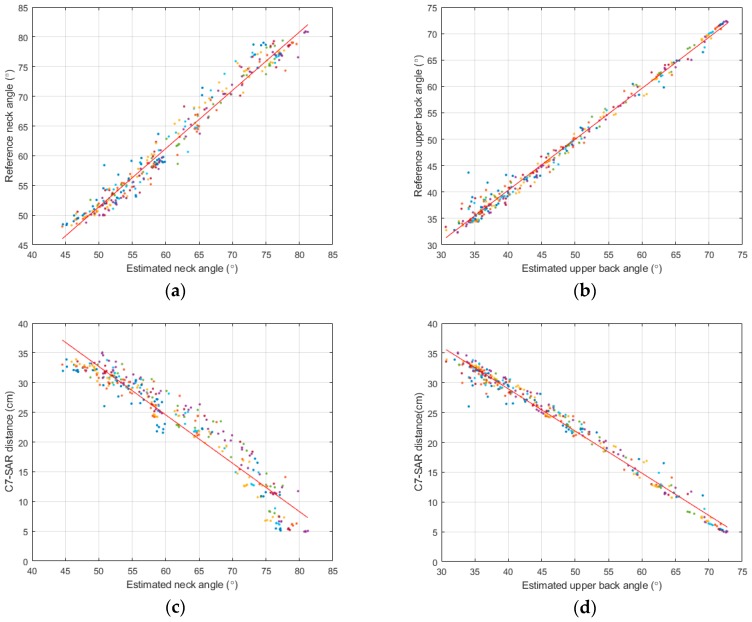
Comparisons of estimated and reference data: (**a**) Estimated vs. reference neck angles. R-square = 0.97; (**b**) estimated vs. reference upper back angles. R-square = 0.99; (**c**) estimated neck angle vs. C7-SAR distance. R-squared = −0.96; (**d**) estimated upper back angle vs. C7-SAR distance. R-squared = −0.99.

Among the 140 walking datasets, five ineffectual sets were discarded due to failures in recording by the optical system. The final plots of 135 walking data segments for seven subjects are shown in Figure 4. Figure 4a,b illustrates the relationship between the estimated flexion angles and the reference data of the neck and upper back, respectively. As can be seen from these figures, the estimated flexion angles have high correlations with the reference angles, since the R-squared values were 0.97 and 0.99 for the neck and back positions, respectively. Additionally, as can be seen in Figure 4a,b, the sensor on the back position tended to perform better than the sensor on the neck as its mean absolute error was 0.9 degrees compared to 1.5 degrees. Furthermore, the data from Figure 4 indicated that the measurements from two sensors can represent the SP angle accurately (Figure 4c,d). The R-squared values were −0.96 and −0.99 for the sensors on the back and neck, respectively, when compared to the C7-SAR distance.

## 4. Discussion

According to the experiment results, there were high correlations between the sensor measurements and the 3D motion camera SP angles. The measurements with acceptable errors for both the neck and upper back positions evidenced that the wearable sensors can be used to estimate the SP in clinical practice. The sensor measurement at the upper back position provides better precision than the neck position, whereas the sensor is more accessible at the neck position than on the back. 

The error of the neck sensor is likely to stem from the asymmetric movements when the subjects’ heads motion is combined with other body parts while walking. Although the PD patient’s head motion is symmetric and coherent with upper limb tremor frequency [27], this motion is unpredictable since PD patients tend to change their neck flexion when the range of view is reduced in the stooped posture. Therefore, neck flexion might not highly associate with trunk flexion as the upper back’s does. However, in contrast to the measurement accuracy, installing a wearable device on the neck seems to be more convenient than on the upper back. NAMU Inc. (Ulsan, Korea) was successful in designing a cervical wearable sensor for neck posture coaching [28], which can measure and provide a reminder when the user is in poor posture. On the upper back position, Upright Go (Upright Technologies, Yehud, Israel) is a popular commercial product with a similar purpose of posture correction [29]. By using adhesive pads, users can freely mount the sensor anywhere on the back.

The result of the analysis induced a prospective method for measuring SP in PD, which can be a non-invasive method for severity assessment in both static and dynamic conditions. This assessment provides a practical result with high accuracy compared to the conventional empirical method. Furthermore, this finding galvanized us to a novel clinical test of evaluating the effect of using a wearable tactile feedback device on Parkinsonism SP treatment as a non-pharmacological approach in daily life. The test was motivated by the fact that PD patients mostly have impaired proprioception which makes them generally not aware of their bending posture [30], and that this postural deformity can be corrected once the patient recognizes it. Compared to SP, kyphoscoliosis is also a deformity of the spine defined by the curvature of the vertebra in the lateral and frontal planes; however, the main difference between SP and kyphoscoliosis is the ability to correct the abnormal posture by effortless activities, such as lying in a supine position, using a high walker or standing against the wall [6]. This ongoing clinical study, which is in progress with real PD patients, shows some satisfactory results. This pilot result suggests that the wearable sensor can be used as a daily life SP monitor for PD.

This study is subject to certain limitations that need to be acknowledged. For instance, the data are simulated from healthy people and the number of subjects involved in the experiments was relatively small. To deal with the problem originated from these limitations, we carefully trained the subjects on how to mimic the PD gait under the supervision of experts. Since PD gait has been studied deeply over decades, its characteristics can be easily modeled and simulated with little effort. That fact was mentioned in Reference [31] where numerous models were proposed to have simulated the PD gait. Another key justification for upholding this notion was described in Reference [32], in which ten healthy elderly subjects successfully mimicked the PD stooped posture. Additionally, with a limited number of subjects, the experimental results in this research still support our claims.

## 5. Conclusions

This study proposed a novel method of measuring SP in PD with a high accuracy. The study also analyzed the tradeoff between the measurement accuracy and the wearing method, and suggested using a wearable sensor as a SP corrector for PD patients in daily life. For that purpose, further clinical study is necessary to investigate our method for the measurement and correction of SP in patients with PD.
